# Are Emerging Non-Thermal Technologies a Viable Solution for Extending the Shelf Life of Rehydrated Cod Products Under Refrigeration?

**DOI:** 10.3390/foods15213907

**Published:** 2026-10-30

**Authors:** Chiara Di Vittori, Avelino Alvarez-Ordóñez, Alberto Vergara, Mercedes López, Márcia Oliveira

**Affiliations:** 1 Postgraduate School of Inspection of Foods of Animal Origin “G. Tiecco”, Department of Veterinary Medicine, University of Teramo, SP 18, 64100 Teramo, Italy; 2 Department of Food Hygiene and Technology, Universidad de León, 24071 León, Spain; 3 Institute of Food Science and Technology (ICTAL), Universidad de León, 24007 León, Spain; 4 Instituto de Investigación Biosanitaria de León (IBIOLEÓN), 24071 León, Spain

**Keywords:** rehydrated cod, non-thermal technologies, seafood preservation, hurdle technology

## Abstract

Cod has long held substantial economic and cultural importance in northern European societies. Today, rehydrated cod, obtained by desalting salted and dried products, is increasingly marketed as a ready-to-cook seafood product but remains highly perishable. The increased water activity, reduced salt content, and structural modifications induced by rehydration can promote microbial growth and quality deterioration, resulting in a limited refrigerated shelf life. This review critically examines available evidence on emerging non-thermal technologies for rehydrated cod, considering how rehydration-induced changes may influence treatment performance. High-pressure processing (HPP), non-thermal atmospheric plasma (NTAP), ultraviolet (UV) radiation, pulsed light, ozonation, and other approaches are evaluated in terms of their antimicrobial effectiveness, effects on quality, advantages, and limitations. Available evidence indicates that HPP is the most extensively investigated technology for delaying microbial spoilage, whereas evidence supporting NTAP, UV radiation, and pulsed light remains limited and does not establish their effectiveness as stand-alone treatments. Hurdle strategies warrant further investigation, although treatment interactions and outcomes depend strongly on matrix properties and processing conditions. Preservation strategies must therefore be optimized and validated directly in rehydrated cod rather than extrapolated from other seafood matrices. Further studies under industrially relevant conditions are needed to establish treatment efficacy and support industrial implementation.

## 1. Introduction

Cod preservation has deep historical and socioeconomic roots in northern Europe. Zooarchaeological evidence from Arctic Norway indicates that dried-fish production was already intensive during the Iron Age and that substantial stockfish surpluses existed during the Viking Age, preceding the emergence of the documented commercial stockfish trade around AD 1200 [1]. Ancient DNA analysis has also traced Viking Age cod bones recovered at the trading settlement of Haithabu to Northeast Arctic cod originating from the Lofoten region, confirming long-distance transport from northern Norway [2]. Together, these findings highlight the historical importance of cod preservation and long-distance trade as sources of food and income for northern European communities during the medieval and early modern periods.

This historical importance persists today, as Atlantic cod (*Gadus morhua*) and Pacific cod (*Gadus macrocephalus*) remain among the most commercially valuable fish species worldwide and are widely appreciated for their nutritional value and culinary versatility. Traditionally, cod is preserved through salting and drying, processes that ensure long-term stability by reducing water activity and inhibiting microbial growth. Before consumption, however, salted cod must be rehydrated through soaking. This step restores moisture and texture, but at the same time it substantially modifies the product’s physicochemical and microbiological characteristics. Rehydration is typically performed by immersing the salted product in chilled water for 24–72 h, with one or more water changes to promote salt removal while restoring tissue hydration [3]. During this process, water content increases markedly, often reaching 70–85%, while salt content decreases to approximately 2–3%. Combined with the structural changes induced by the preceding salting and drying processes, these changes contribute to further modifications in muscle structure and composition. Although salting effectively inhibits microbial growth, the desalting process creates conditions that favor microbial proliferation, making the product highly perishable [4]. Previous studies have shown that the microbial communities associated with rehydrated cod are influenced by the selective pressure exerted by salting and drying, which may result in spoilage dynamics that differ from those typically observed in fresh fish [5]. Nevertheless, rehydrated cod is particularly susceptible to spoilage during refrigerated storage, even when vacuum packaging is applied. This challenge has become even more relevant with the increasing industrialization of the rehydration process, driven by the growing demand for ready-to-cook seafood products. Under these conditions, ensuring both microbiological safety and product quality represents a major concern in modern seafood processing.

Conventional food preservation methods often rely on high temperatures to achieve microbial inactivation; however, thermal treatments may induce undesirable changes in organoleptic and functional properties [6]. These effects are particularly relevant to rehydrated cod, in which high water activity and rehydration-induced changes in muscle structure make microbial control essential while preserving texture and overall quality. This has prompted interest in non-thermal preservation strategies as potential alternatives.

The emerging non-thermal technologies that have received more attention as potential alternatives include high-pressure processing (HPP), non-thermal atmospheric plasma (NTAP), pulsed electric fields (PEF), ultraviolet light (UV), ultrasound (US), pulsed light (PL), and ozone treatments [7]. These approaches allow effective microbial inactivation at near-ambient temperatures while minimizing thermal degradation of food components [8]. In seafood, they have been widely studied in fresh and minimally processed products, where promising microbial inactivation effects have been reported alongside a good preservation of sensory and nutritional attributes. However, their application to rehydrated products remains relatively limited.

The characteristics of rehydrated products may significantly influence the effectiveness of these technologies. Increased water content, structural modifications, and changes in microbial ecology can affect key factors such as mass transfer, penetration of reactive species, and tissue susceptibility to pressure or oxidative stress. As a result, responses observed in fresh or frozen products cannot always be directly extrapolated to rehydrated matrices. Despite the growing interest in non-thermal technologies for seafood processing, the lack of matrix-specific studies still limits process optimization.

The current review examines the challenges and opportunities associated with the application of emerging non-thermal technologies to seafood products, with particular emphasis on rehydrated cod. It focuses on how rehydration-induced changes influence treatment effectiveness, product quality, and shelf life, while also considering the potential of hurdle strategies. Finally, current limitations and future research needs are outlined.

## 2. Rehydration as a Critical Processing Step

The rehydration of salted fishery products is driven by concentration and osmotic gradients between the salted muscle and the surrounding medium, promoting the transfer of water, salt, and soluble proteins through diffusion and osmotic mechanisms. In addition, capillary forces and structural expansion drive hydrodynamic flows within the porous muscle structure [3]. As a result, the desalting of salted cod represents a critical processing step that induces significant physicochemical, structural, and microbiological changes. The muscle matrix undergoes substantial modifications that affect salt and water distribution, protein functionality, and the stability of the final product [3,9].

The commercial process of rehydration generally starts from salted or dried-salted cod, which is portioned and immersed in chilled water for 24–72 h, depending on product thickness and the desired final salt content. The soaking water is renewed one or more times to maintain the concentration gradient and promote salt removal and water uptake. After rehydration, the product is drained, packaged, and stored under refrigeration. Processing time, temperature, water-to-fish ratio, water-renewal frequency, and portion geometry therefore influence both desalting kinetics and the characteristics of the final product [3,10].

### 2.1. Physicochemical Changes During Rehydration

The rehydration of salted cod is a process governed by mass transfer phenomena, involving water uptake and salt leaching from the muscle tissue. Consequently, the water content of cod increases while its salt levels decrease, leading to a progressive restoration of the original muscle hydration state.

Fidalgo et al. [9], for example, reported that salt content decreased from approximately 18% to 1.5% at 20 °C and 2.5% at 4 °C after 16 h of desalting, illustrating the influence of processing temperature on salt-removal kinetics.

However, the extent and rate of these changes also depend on the previous salting history. Before the main dry-salting stage, cod may undergo pre-salting by brining, brine injection followed by immersion, or pickle salting. Initial brining commonly lasts 1–4 days, and its concentration and duration determine the extent of salt uptake and water exchange before subsequent dry salting. In cod loins, increasing brine concentration from 6% to 24% accelerated salt diffusion and water loss, whereas lower concentrations produced greater initial weight gain; notably, the highest effective salt diffusion was observed at 15% rather than at 18% or 24% [11]. Moreover, varying the initial brine concentration did not significantly affect the yield, chemical composition, or water-holding capacity of the final salted and rehydrated product. However, better overall quality was reported at 16° Baumé (17.5% NaCl) than at 20 or 24° Baumé (22.5% and 25.6% NaCl, respectively) [12]. These findings indicate that the effects of brine concentration are not necessarily linear and that both the concentration and duration of pre-salting must be considered in relation to the subsequent salting stage and rehydration. During rehydration, this two-way exchange, involving sodium chloride (NaCl) loss and water uptake, leads to an increase in product weight and volume [10]. These changes also affect the composition and functional properties of muscle components. In particular, the decrease in NaCl concentration promotes the solubilization of proteins previously stabilized under high ionic strength conditions, resulting in their loss into the soaking medium and a reduction in total soluble protein content [3,13,14]. Remarkably, key properties such as the isoelectric point, water-holding capacity, and protein functionality are significantly influenced by factors including raw material composition, salting method, sample geometry, and processing conditions such as temperature [7,10].

### 2.2. Structural and Textural Modifications

The structural integrity of cod muscle changes markedly during salting and subsequent rehydration. Increasing salt concentration promotes water loss, protein denaturation, and aggregation, contributing to tissue compaction and increased hardness [15]. During desalting, salt loss and water uptake instead promote swelling and progressive softening. The salt–hardness profile is non-linear: the rapid initial loss of salt is accompanied by the greatest reduction in hardness, whereas both salt removal and softening progressively slow as equilibrium is approached, producing a plateau-like trend [9,16]. Recent three-dimensional imaging also confirms that desalting is accompanied by progressive changes in product volume [17]. However, the original muscle structure is not completely restored because some salting-induced changes are irreversible.

Pre-salting history also influences the structural response of cod muscle during subsequent dry salting, and these effects may persist after rehydration. Although muscle salt content exceeded 20% NaCl in all the procedures examined, injection followed by brining resulted in lower protein aggregation and greater water retention than brining alone or pickle salting, with protein aggregation increasing in the order injection and brining < brining < pickling [18]. Therefore, the properties of rehydrated cod depend not only on its final salt content but also on the pre-salting procedure and its effects on muscle protein structure.

### 2.3. Biochemical and Enzymatic Changes

Rehydration also affects the biochemical stability of cod, particularly through changes in endogenous enzyme activity and protein oxidation. The activity of enzymes such as cathepsins and phosphatases has been reported to decrease during desalting, probably due to the loss of soluble proteins into the desalting water, whereas lipase activity may increase, potentially contributing to lipid degradation processes [9].

In addition to these processing-related changes, the biochemical and enzymatic characteristics of cod muscle vary with fish size, although the direction of this relationship depends on the enzyme considered. In Atlantic cod muscle, the maximal activities of the glycolytic enzymes phosphofructokinase and lactate dehydrogenase increased with body size, whereas those of cytochrome c oxidase and nucleotide diphosphate kinase decreased [19]. These allometric differences describe the metabolic characteristics of the raw muscle and should be distinguished from the effect of portion thickness during processing. During desalting, thinner cod pieces reached salt and water equilibrium more rapidly and showed faster diffusion of water, NaCl, and soluble proteins than thicker pieces [20]. Salting history is also relevant because chymotrypsin-, trypsin-, collagenase-, and elastase-like activities increased during the first five days of salting but subsequently declined as curing progressed, whereas acidic hemoglobin-hydrolyzing activity decreased with increasing salt concentration in the muscle [21].

Therefore, fish size may influence the initial enzymatic profile of cod muscle, while portion thickness and the evolution of salt concentration affect subsequent mass-transfer and proteolytic processes.

At the same time, relevant protein changes such as the leaching of water-soluble proteins and the formation of cross-linked aggregates, often associated with oxidative processes, may occur. These modifications, linked to sulfhydryl oxidation and partial protein unfolding, can affect the functional and sensory characteristics of the product [14].

Overall, these alterations may contribute to quality deterioration, although the relative contribution of each mechanism remains unclear and requires further investigation.

### 2.4. Microbial Implications of Rehydration

The rehydration process has a profound impact on microbial dynamics, as increased water activity and reduced salt concentration favor rapid microbial growth. Several studies have reported significant increases in spoilage-associated microorganisms during desalting and subsequent storage. These include bacterial populations capable of producing ammonia, biogenic amines, hydrogen sulfide and other volatile spoilage compounds, such as members of the genera *Pseudomonas* and *Psychrobacter* [5,22,23]. Temperature is a key factor during cod desalting, as higher temperatures can accelerate microbial proliferation and spoilage. For instance, desalting at temperatures above 10–15 °C has been associated with off-flavor development and product rejection [24].

Rehydration is performed at both household and industrial levels. In households, salted cod is traditionally immersed in stagnant water at either room temperature or under refrigeration. Industrial rehydration follows similar principles but generally requires 2–3 days, depending on the thickness of the fish portions, and involves several water changes to promote mass transfer [10,25]. However, household practices can vary considerably in terms of duration, temperature, and water replacement frequency, potentially affecting microbiological safety. Pedro et al. [26] found lower bacterial loads in cod desalted for 24 h at 3 °C than in samples at 20 °C. At the higher temperature, *Staphylococcus aureus* counts could reach approximately 6 log CFU/g, highlighting the potential risk of enterotoxin production under inadequate temperature control.

From a food-safety perspective, salt removal and the associated increase in water activity may create conditions conducive to pathogen growth if contamination is present. *Vibrio alginolyticus* was among the Gram-negative bacteria isolated from soaked cod, suggesting that potentially pathogenic microorganisms surviving in salted products may represent a hazard during rehydration, particularly when the product is consumed raw or insufficiently cooked [5]. Nevertheless, a more recent investigation of industrially processed soaked cod reported no detection of *Listeria monocytogenes*, *Escherichia coli*, *Salmonella* spp., or *Vibrio* spp. [27]. Thus, the available evidence does not demonstrate the consistent occurrence or proliferation of pathogenic *Vibrio* spp. in rehydrated cod. However, prolonged rehydration and inadequate temperature control may favor pathogen growth if contamination occurs. Refrigerated rehydration and hygienic handling are therefore essential to minimize potential food-safety risks.

In parallel, the soaking process modifies the microbial composition of salted cod. While Gram-positive cocci, particularly *Staphylococcus* spp., dominate the microbiota of salted and dried-salted products, rehydration favors the growth of Gram-negative psychrotrophic bacteria. In particular, *Pseudomonas* spp. have been reported as predominant members of the microbiota of soaked cod, together with other spoilage-associated bacteria such as *Shewanella putrefaciens* in freshly rehydrated products. As a result, the microbiota becomes more similar to that of fresh fish [5].

In addition to the increase in water activity and reduction in salt concentration, the structural disruption of muscle tissue and the associated biochemical changes may enhance microbial activity by increasing the availability of substrates for bacterial metabolism. These factors collectively make rehydrated cod highly perishable, highlighting the limitations of conventional preservation approaches. In this context, non-thermal technologies have emerged as promising alternatives for microbial control while preserving product quality.

Available evidence suggests that specific spoilage organisms respond differently to non-thermal treatments. In fresh mackerel, NTAP reduced both *Pseudomonas* spp. and lactic acid bacteria within 24 h of treatment [28], whereas in gilthead sea bream, it reduced *Pseudomonas* spp. but had no effect on lactic acid bacteria [29]. Similarly, studies on sous-vide cod suggest that HPP may alter the composition of the surviving microbial community [30]. However, these findings derive from fresh or cooked fish products and cannot be directly extrapolated to raw rehydrated cod. To date, studies specifically characterizing spoilage organisms and their recovery following non-thermal treatment in this matrix remain unavailable.

## 3. Emerging Non-Thermal Technologies Applied to Rehydrated Cod

Non-thermal preservation technologies may extend the shelf life of fishery products while limiting heat-induced quality changes [31]. Their effectiveness in rehydrated cod, however, requires matrix-specific evaluation because rehydration restores high water activity while the product retains residual salt and persistent structural and biochemical modifications induced by salting and drying. The evidence reviewed in the following sections can be divided into direct matrix-specific evidence and indirect evidence extrapolated from other seafood products. Direct evidence of microbial control and shelf-life extension in rehydrated cod is currently available mainly for HPP, whereas studies on PEF have focused on rehydration kinetics and mass-transfer enhancement rather than microbial preservation. For NTAP, PAW, UV-C, pulsed light, ozone, ultrasound, and ionizing irradiation, the potential application to rehydrated cod is largely inferred from studies conducted on fresh, frozen, smoked, or dried seafood. This distinction is important because rehydrated cod combines high water activity and residual salt with irreversible structural and biochemical modifications induced by salting and drying, as well as a distinct spoilage microbiota. These characteristics may influence microbial resistance, treatment penetration, post-treatment recovery, and quality deterioration. The available literature does not establish whether the scarcity of matrix-specific studies reflects limited industrial adoption or the historical concentration of research on other seafood products. Nevertheless, it currently prevents the effectiveness of these technologies in rehydrated cod from being established without direct experimental validation.

A summary of the main available non-thermal technologies, their mechanisms of action, advantages, and limitations is provided in Table 1.

### 3.1. High-Pressure Processing (HPP)

HPP is a non-thermal preservation technology based on the application of isostatic pressures (400–600 MPa) for short times (commonly from 1.5 to 6 min), enabling the inactivation of vegetative microorganisms while preserving the sensory and nutritional quality of foods [39]. Since HPP is usually applied to products in their final packaging, it minimizes the risk of post-processing contamination and contributes to maintaining microbiological safety during storage [39]. Its effectiveness depends on several factors, including intrinsic product characteristics such as water activity and pH, as well as processing parameters (pressure level and holding time) and inherent microbial resistance [39]. In rehydrated cod, high water activity is compatible with effective HPP-mediated microbial inactivation, whereas residual salt and other matrix characteristics may modify microbial resistance. At the same time, salt reduction removes an important inhibitory hurdle and may facilitate the recovery of sublethally injured cells during subsequent storage.

Among the available technologies, HPP is currently the only approach for which direct evidence is available in rehydrated cod. However, the reported shelf-life extensions vary substantially and cannot be interpreted as directly comparable outcomes without considering differences in experimental design. Rode and Rotabakk [4] found that treatment at 600 MPa for 5 min maintained low bacterial counts for at least 49 days at 4 °C, whereas counts increased significantly after 15 days following treatment at 400 or 500 MPa. In that study, clipfish and saltfish differed in portion size, rehydration time, water-to-fish ratio, water renewal, and packaging conditions. By contrast, Fidalgo et al. [9] applied 400 or 550 MPa for 5 min after a 16 h desalting process conducted at 4 or 20 °C and reported a shelf life of 14–21 days, compared with approximately 7 days for untreated samples. Ferri et al. [27] investigated industrially processed *Gadus macrocephalus* fillets vacuum skin-packaged in films with different oxygen transmission rates and treated at 600 MPa for 5 min; despite the marked initial microbial reduction, bacterial loads could still exceed 6 log CFU/g after 35 days at 4 °C.

Accordingly, the reported range of 14–49 days cannot be attributed to pressure level alone. Fish species and raw-material history, desalting conditions, residual salt content, initial microbial load, packaging permeability, storage design, and the microbiological or sensory criteria used to define shelf life are all potential sources of variability. Although the studies used untreated controls and statistical analyses, differences in their sampling structures and replication schemes, together with the absence of harmonized protocols and shelf life criteria, prevent a direct quantitative synthesis of their findings. Taken together, the available evidence consistently indicates that HPP can improve the microbial stability of rehydrated cod, particularly at higher pressures, but does not support a universal shelf life extension that can be transferred across products and processing conditions.

Evidence from other fish matrices also indicates that HPP efficacy may be enhanced by complementary preservation hurdles. In raw and smoked trout, treatment at 200 MPa for 15 min combined with 0.5% liquid smoke and prior freezing reduced *L. monocytogenes* by 1.93 and 5.48 log CFU/g, respectively, illustrating both the potential of combined treatments and the strong influence of the product matrix [40].

Beyond microbial inactivation, HPP can induce important physicochemical and biochemical changes in cod muscle. Increased protein solubility and peptide release, improved digestibility, and reduced allergenicity compared with thermal treatments have been reported in fish products [41]. However, the extent to which these effects apply specifically to rehydrated cod remains insufficiently established. Pressure can also affect muscle structure and quality attributes such as texture, color, and water-holding capacity. Evidence from other seafood matrices confirms that these quality responses are product-dependent. Treatments at 400–600 MPa for 10 min altered whitening and water mobility in thawed deep-water rose shrimp, gray mullet, and tiger prawn, while effects on protein denaturation, protein solubility, texture, and lipid oxidation varied among species [42].

Rode and Rotabakk [4] reported that processing at 600 MPa resulted in greater fluid release immediately after treatment (5.6 ± 1.2%) than at 400 or 500 MPa (3.3 ± 1.0%), while also increasing product firmness. Conversely, color changes were limited compared with those commonly observed in fresh cod, possibly because salting and drying had already modified pressure-sensitive muscle proteins. These findings demonstrate that stronger microbial control at higher pressures must be balanced against pressure-induced quality changes and that responses observed in fresh fish cannot be transferred directly to the rehydrated matrix.

Additional limitations must also be considered. Lipid oxidation may be enhanced at pressures above 400 MPa because of the release of pro-oxidant metal ions, such as iron and copper, which catalyze oxidative reactions. Mitigation strategies reported in other muscle foods include optimization of pressure level and holding time, vacuum packaging, oxygen scavengers, and antioxidant-active packaging [43]. However, these findings cannot be transferred directly to rehydrated cod, and existing matrix-specific studies have not evaluated these approaches specifically against HPP-induced lipid oxidation. Future studies should therefore compare pressure–time combinations and oxygen-control or antioxidant strategies while simultaneously monitoring microbial inactivation, lipid oxidation, and sensory quality. Pressure-induced changes in pH are generally limited but may also reflect microbial activity and metabolite formation during storage.

Despite its demonstrated potential, the industrial application of HPP to rehydrated cod remains constrained by high capital investment and equipment maintenance costs. Moreover, microbial recovery during storage raises concerns about long-term product stability, particularly under suboptimal cold chain conditions. Beyond microbial regrowth, HPP may also influence the composition of the residual microbiota. Although evidence for raw rehydrated cod is still limited, studies on HPP-treated vacuum-packed cooked cod products suggests that pressure treatment can induce shifts in microbial community structure during storage [30]. Whereas untreated samples were characterized by the succession of *Pseudomonas* and *Enterobacteriaceae,* HPP-treated samples showed a predominance of *Aerococcus* and *Enterococcus* [30]. Nevertheless, whether similar microbial shifts occur in raw rehydrated cod remains to be determined.

Overall, HPP is currently the most substantiated non-thermal preservation technology for rehydrated cod, although the available evidence is insufficient to establish a universally optimal treatment. Further matrix-specific studies using standardized processing conditions, storage protocols, and acceptability criteria are required to determine whether HPP alone can ensure adequate preservation or whether its effectiveness would benefit from combination with additional preservation hurdles.

### 3.2. Non-Thermal Atmospheric Plasma (NTAP)

NTAP, also known as cold atmospheric plasma (CAP), is an emerging non-thermal technology generated at ambient temperature and atmospheric pressure when an electrical discharge is applied to a gas, causing ionization, dissociation and excitation of its atoms and molecules [44]. NTAP contains a complex mixture of reactive chemical species, including reactive oxygen and nitrogen species (ROS and RNS), charged particles, free radicals, atoms and molecules in the excited or non-excited state [45]. These components interact with food components and microbial cells, causing oxidative damage and leading to their inactivation. Operating at near-ambient temperature and atmospheric pressure, NTAP is particularly suitable for the surface decontamination of heat-sensitive foods [46]. Its antimicrobial efficacy is primarily associated with membrane disruption, protein oxidation, and interference with essential cellular functions [46]. However, the antimicrobial activity of directly applied NTAP is largely restricted to exposed surfaces because of the limited penetration of plasma-generated reactive chemical species into solid food matrices. Consequently, microorganisms located within the muscle tissue may be less affected by the treatment.

In rehydrated matrices, the high moisture content may facilitate the diffusion of plasma-generated reactive species across exposed surfaces, potentially enhancing antimicrobial activity. At the same time, water, proteins, lipids, and soluble compounds released during rehydration may react with or quench these species, thereby reducing the effective plasma dose reaching microbial cells and promoting oxidative reactions in the product. The altered muscle microstructure may also expose microorganisms located in superficial tissue layers while continuing to protect cells situated within crevices or deeper regions, where the penetration of reactive species is limited.

The available seafood studies are methodologically heterogeneous and show that NTAP effectiveness depends strongly on plasma systems, treatment conditions, the target microorganisms, and the product matrix. Albertos et al. [28] applied in-package dielectric barrier discharge plasma to fresh mackerel fillets at 70 or 80 kV for 1–5 min. Reductions of up to approximately 2 log CFU/g were reported for *Pseudomonas* spp. and psychrotrophic lactic acid bacteria, whereas total aerobic mesophilic counts were not significantly reduced, indicating that microbial groups within the same product may differ in susceptibility. In smoked salmon inoculated with foodborne pathogens, Colejo et al. [47] applied a laboratory-scale atmospheric plasma jet operated with air at a flow rate of 10 L/min and a voltage difference of 2 kV for 15 min, obtaining reductions of only 0.5–1.3 log CFU/g, although no significant sensory changes were detected. The relatively limited inactivation may reflect the protective effects of the smoked-salmon matrix and the restricted penetration of plasma-generated species. By comparison, Wang et al. [32] treated inoculated tilapia fillets at 70 kV for 60–300 s and reported reductions of 2.34 log CFU/g for *Salmonella* Enteritidis and 1.69 log CFU/g for *L. monocytogenes* after 300 s, showing a treatment-time-dependent effect.

Spanou et al. [29] evaluated four in-package treatments on fresh gilthead sea bream stored at 2 °C. The least intensive condition, 5 min at 1.0 kHz, resulted in a shelf life of 9 days, whereas 10 min at 1.5 kHz extended it to 12 days. After 16 days, the maximum reductions relative to untreated controls were 1.49, 1.24, and 1.43 log CFU/g for total mesophilic counts, total psychrotrophic counts, and *Pseudomonas* spp., respectively. However, the effect on *Enterobacteriaceae* was limited, and no effect was observed against lactic acid bacteria. These findings demonstrate that an increase in treatment intensity may improve the control of selected spoilage populations but does not produce an equivalent response across all microbial groups.

The reported quality effects are similarly inconsistent. In fresh gilthead seabream (*Sparus aurata*) fillets, Spanou et al. [29] observed no relevant changes in color or texture, but lipid oxidation increased by up to 75.5% under the most intensive treatment conditions. In tilapia fillets treated with high-voltage atmospheric cold plasma at 70 kV for 60–300 s, Wang et al. [32] found that thiobarbituric acid-reactive substances increased significantly, particularly after 180 s, while the longest treatment (300 s) reduced redness and immobilized water content and increased free water content. By contrast, Pérez-Andrés et al. [48] found no significant increase in lipid oxidation in mackerel treated at 80 kV for 5 min, although protein carbonyl formation was accelerated during storage. Moreover, the study by Liu et al. [49] focused specifically on lipid oxidation kinetics in tilapia and therefore provides evidence of potential quality deterioration rather than direct evidence of microbial inactivation or shelf life extension. Evidence from other animal-derived foods also illustrates this dose–quality trade-off. In pork, NTAP treatments longer than 3 min reduced total viable counts and *Pseudomonas* spp., whereas the most prolonged treatment (9 min) increased lipid oxidation and affected color [50]. Although these findings cannot be transferred directly to seafood, they reinforce the need to evaluate microbial efficacy and quality changes simultaneously for each specific food matrix.

Overall, the apparent differences among studies may be related to fish composition, surface structure, initial contamination, target microorganisms, plasma-generation system, treatment voltage and frequency, exposure time, packaging atmosphere, and storage conditions. Consequently, evidence obtained from fresh mackerel, sea bream, tilapia, or smoked salmon can only be used to formulate hypotheses for rehydrated cod. Its high-water activity, residual salt content, salting-induced protein modifications, altered microstructure, and surface exudate may modify both microbial inactivation and oxidative responses. In the absence of direct studies, NTAP cannot yet be considered an established standalone preservation treatment for rehydrated cod. Its limited penetration, possible effects on sensory characteristics during prolonged treatments, and potential formation of undesirable reaction products also require specific safety and regulatory assessment before commercial application. In addition to direct treatments, NTAP can also be used to generate plasma-activated water (PAW), an indirect approach through exposure of water to plasma, resulting in the formation and diffusion of new ROS and RNS responsible for its antimicrobial activity [51,52]. Plasma-activated water (PAW) may represent a particularly interesting approach for rehydrated cod, as the product already undergoes prolonged immersion in water during the desalting process. The use of PAW as a rehydration medium could combine desalting and microbial decontamination in a single processing step, thereby reducing microbial loads before packaging and storage. Furthermore, the use of PAW during desalting may allow reactive species to be distributed throughout the soaking medium and reach areas of the product that are less accessible to the ionized gas plasma. Although this application has not yet been investigated in rehydrated cod, promising results obtained with PAW in other food matrices suggest that it may warrant further research. Particular attention should be given to its effects on microbial inactivation, oxidative stability, and quality attributes during desalting and subsequent refrigerated storage.

Despite their promising antimicrobial potential, the application of NTAP and PAW to rehydrated cod remains largely unexplored and requires further investigation. Major limitations include the lack of matrix-specific data, the risk of oxidative damage, and the need for improved process standardization before industrial implementation can be considered.

### 3.3. Ultraviolet Light (UV)

Ultraviolet (UV-C, 200–280 nm) radiation is a non-thermal decontamination technology capable of inactivating a broad spectrum of microorganisms, primarily through nucleic acid damage and the formation of DNA photoproducts such as cyclobutane pyrimidine dimers, which interfere with replication and lead finally to cell death [53]. This residue-free technology requires relatively low energy input and mild processing conditions, making it attractive for food applications. However, its low penetration depth limits its effectiveness to surface decontamination [54]. This may pose a barrier to its use in rehydrated cod, where microbial contamination may also occur within the muscle due to the increased water mobility during desalting.

UV-C treatment has shown potential to reduce microbial loads and delay spoilage in seafood during refrigerated storage. Tandel et al. [33] evaluated UV-C exposure for 20, 40, and 60 s in reef cod fillets stored at 4 ± 1 °C for 15 days. After 10 days, fillets treated for 20 s had total plate counts of 5.2 log CFU/g, compared with 6.5 log CFU/g in untreated samples, while maintaining sensory acceptability and structural integrity for up to 10 days. These findings suggest that shorter exposure provides greater overall preservation, highlighting that extending UV-C treatment does not necessarily improve efficacy when microbial control and product quality are considered together. However, direct comparison among studies is hindered by substantial differences in UV dose, source-to-product distance, exposure time, surface geometry, initial microbial load, packaging conditions, and the criteria used to assess spoilage and shelf life. Findings from dried fish are particularly difficult to extrapolate to rehydrated cod, as differences in water activity and surface structure may influence microbial resistance and UV penetration [55]. To date, the effectiveness of UV-C has not been directly investigated in rehydrated cod. Given its limited penetration, UV-C may reduce microbial contamination on exposed surfaces, but its effectiveness against microorganisms within the water-rich, structurally altered muscle remains uncertain. Therefore, UV-C should currently be considered a complementary surface decontamination treatment rather than a standalone preservation strategy for rehydrated cod.

### 3.4. Other Emerging Non-Thermal Technologies

In contrast to the technologies discussed above, the following approaches have received comparatively limited attention as alternative non-thermal technologies for fish and seafood, particularly in rehydrated matrices [56]. Because they differ in their primary technological function, they are discussed in two groups: technologies evaluated mainly for microbial decontamination and shelf-life extension, including ozone, pulsed light, and ionizing irradiation; and technologies investigated primarily for process intensification or structural modification, including ultrasound and pulsed electric fields.

#### 3.4.1. Preservation-Oriented Technologies

Ozone is a strong oxidizing agent widely investigated in food systems due to its ability to inactivate microorganisms through oxidative damage to cellular components. In seafood, ozone has been mainly applied to fresh fish and shellfish [34,57,58], where it has demonstrated effectiveness in reducing microbial loads and delaying spoilage. In vacuum-packaged rainbow trout stored at 4 ± 0.5 °C, aqueous ozonation at 0.6 or 0.4 mg/L for 60 or 90 min, respectively, delayed the growth of mesophilic aerobic bacteria, *Pseudomonas* spp., H_2_S-producing bacteria, *Brochothrix thermosphacta*, lactic acid bacteria, and *Enterobacteriaceae*. Shelf life reached 10 and 12 days following the 60 and 90 min treatments, respectively, although these prolonged exposure times may limit industrial applicability [34]. However, ozone may also promote lipid oxidation, potentially compromising product quality. In rehydrated cod, the high water activity may favor ozone dissolution at exposed surfaces, whereas organic matter released during rehydration may rapidly consume ozone and limit its penetration. These matrix characteristics could therefore reduce antimicrobial efficacy while also increasing the risk of oxidative degradation of lipids and proteins.

PL employs high-intensity, short-duration pulses of broad-spectrum light (200–1100 nm), including ultraviolet, visible, and infrared wavelengths, to achieve microbial inactivation on food surfaces [59]. Its antimicrobial action is mainly attributed to photochemical and photothermal effects that damage cellular components, leading to microbial cell death [59]. PL has been widely investigated as a rapid and effective method for surface decontamination of foods and food contact materials, supporting the concept of minimal processing [59]. In Atlantic salmon, PL treatment at 2280 V for 15 s reduced *Pseudomonas fragi* by 99.21% and extended refrigerated shelf life at 4 °C from 3 to 7 days, primarily by inhibiting *Pseudomonas* rather than *Photobacterium* [60]. In large yellow croaker, treatment with 30 pulses at 300 J/pulse achieved an initial microbial reduction of 86.27 ± 4.32% and extended shelf life by 2–3 days at 4 °C. However, increasing the intensity to 500 J/pulse promoted lipid oxidation and produced sulfurous and metallic odors, demonstrating a dose-dependent trade-off between microbial control and quality [35]. Ionizing irradiation is a preservation technology based on the treatment of food with high-energy radiation (e.g., gamma rays, X-rays, or electron beams) to inactivate microorganisms and improve food safety, primarily through the induction of ionization processes that damage cellular components and DNA [61]. It has demonstrated microbial inactivation capacity, as well as the ability to extend the shelf life of seafood products while maintaining quality attributes [62]. Nevertheless, its application in seafood remains constrained by regulatory requirements, consumer acceptance, and possible dose-dependent quality changes. In stripped weakfish fillets irradiated at 1.5, 4, or 6.5 kGy and stored at 4 ± 1 °C, microbial reductions were dose-dependent. Treatment at 4 kGy reduced the initial mesophilic aerobic population by approximately 4.5 log cycles and extended shelf life from 3 to 19 days while maintaining sensory acceptability [63]. In Atlantic cod, electron-beam treatments of 2–10 kGy produced dose-dependent changes in color, texture, protein oxidation, and lipid oxidation, with 4 kGy proposed as the most appropriate compromise between treatment efficacy and product quality [38].

#### 3.4.2. Process-Intensification and Complementary Technologies

US, based on acoustic cavitation, has also been investigated in seafood processing for its ability to induce physicochemical modifications and enhance mass transfer [64]. When applied alone, its antimicrobial effectiveness is limited, with only modest reductions in microbial loads reported in fish fillets, particularly in white fish species such as cod [36]. In contrast, studies on Atlantic cod indicate that US can modify protein structure and improve digestibility, highlighting its greater potential for enhancing functional and nutritional properties rather than extending shelf life [37].

PEF technology is based on the application of short, high-voltage pulses to food placed between electrodes, inducing electroporation of cell membranes and increasing their permeability. Although PEF has been extensively investigated for microbial inactivation, this application has been most extensively studied in liquid foods, whereas in solid matrices such as fish muscle PEF is more commonly used to enhance mass transfer and modify tissue structure [65]. PEF has been investigated in cod processing; however, its application has been primarily limited to the enhancement of mass transfer during the rehydration of dry-salted products, where electroporation facilitates water uptake and salt removal [10].

Overall, these technologies are supported by different types and levels of evidence and should not be considered equivalent preservation options. Evidence that ultrasound or pulsed electric fields improve desalting or mass transfer cannot be considered direct evidence of antimicrobial preservation. Moreover, differences in fish species, product state, treatment intensity, packaging, storage conditions, and microbiological or quality endpoints prevent a quantitative comparison among the available studies. For rehydrated cod, ozone and ionizing irradiation may provide microbial control but raise concerns related to oxidation, quality changes, regulation, or consumer acceptance, whereas pulsed light remains limited to exposed surfaces. Ultrasound and pulsed electric fields may therefore be more appropriately considered as pre-treatments or process-intensification tools within hurdle strategies rather than as primary antimicrobial interventions. Direct comparative studies in rehydrated cod are required before the relative effectiveness of these technologies can be established.

#### 3.4.3. Additional Emerging Preservation and Bioprotective Approaches

Dense-phase carbon dioxide (DPCD) uses pressurized CO_2_ under subcritical or supercritical conditions to inactivate microorganisms and enzymes through CO_2_ dissolution, intracellular acidification, and membrane disruption [66]. In raw salmon, increasing pressure from 150 to 250 bar enhanced the inactivation of *S. aureus* and reduced D-values from 41.67 to 26.31 min, although color changes were observed [67]. However, its application to solid foods is generally more challenging than its application to liquids because CO_2_ transfer within the matrix may limit treatment efficiency. DPCD has not yet been evaluated in rehydrated cod.

Electrolyzed water (EW) contains reactive chlorine species and can be applied as a washing medium or converted into electrolyzed ice. Slightly acidic EW ice reduced microbial growth and delayed physicochemical and sensory deterioration in squid compared with conventional ice [68]. More recently, slightly acidic EW combined with nanobubbles reduced the initial total viable count and delayed microbial and quality deterioration in vacuum-packed large yellow croaker stored at 4 °C, extending shelf life by approximately two days [69]. However, because the technologies were applied in combination, their individual contributions to shelf life extension cannot be established. The incorporation of EW into desalting water or post-desalting rinses could represent a complementary approach for rehydrated cod, although the effects of organic matter, residual salt, active-chlorine stability, and possible oxidative changes require matrix-specific evaluation.

Oscillating magnetic fields (OMFs) have been investigated mainly as an aid to supercooling rather than as a standalone microbial-inactivation treatment. In Atlantic salmon, a supercooling system assisted by both OMFs and PEFs maintained lipid stability and drip loss while producing lower microbial counts than refrigerated or frozen storage [70]. However, because the technologies were applied together, the contribution of OMFs alone cannot be established.

Bioactive or bioprotective coatings provide a physical barrier while delivering antimicrobial or antioxidant compounds. A chitosan–alginate coating containing fucoidan extended the refrigerated shelf life of rainbow trout fillets from 6 to 16 days and reduced microbial growth, lipid oxidation, and total volatile basic nitrogen accumulation [71]. Although these results demonstrate their potential as complementary hurdles, coating adhesion, release kinetics, sensory effects, and performance on the moist surface of rehydrated cod require direct validation.

## 4. Hurdle Strategies and Process Optimization

The high perishability of rehydrated cod, driven by its elevated water activity, reduced salt content, and structurally altered muscle matrix, makes effective preservation particularly challenging. In this context, achieving substantial shelf life extension through a single preservation factor may require the application of more intensive treatments, potentially increasing the risk of undesirable changes in product quality. In such systems, microbial growth and spoilage processes are influenced by multiple interacting factors, highlighting that effective preservation strategies should rely on the combination of complementary hurdles rather than on individual treatments. Accordingly, the concept of hurdle technologies provides a useful framework, as it integrates different preservation factors to achieve enhanced microbial stability while maintaining product quality. Within this framework, ultrasound and pulsed electric fields may act as enabling pre-treatments by modifying tissue structure or mass transfer, potentially influencing the effectiveness of a subsequent preservation treatment.

Despite its theoretical relevance, to date, evidence of the application of combined preservation approaches in rehydrated cod remains extremely limited. The work of Rode and Rotabakk [4] represents one of the most relevant contributions, evaluating the combination of HPP with different packaging conditions, including vacuum, modified atmosphere packaging (MAP), and carbon dioxide (CO_2_) emitters. While HPP alone proved highly effective in controlling microbial growth and extending shelf life up to 49 days under refrigeration, MAP or the addition of CO_2_ emitters did not significantly improve the final quality outcomes. These results do not undermine the potential of hurdle strategies in cod preservation but rather highlight that their effectiveness must be validated, as it is strongly matrix-dependent, and may not always result in additive or synergistic effects. In the case of rehydrated cod, the inclusion of additional preservation hurdles does not necessarily improve performance, and their impact appears to be constrained by product-specific properties such as gas diffusion, water activity, and microbial ecology. An additional aspect to consider is the presence of sublethally injured microbial populations following non-thermal treatments, as their recovery strongly depends on matrix conditions such as water activity, oxygen availability, and nutrient diffusion.

Given the limited number of studies specifically focused on rehydrated cod, evidence from other fish products can provide only indirect support for generating hypotheses about combined preservation treatments.

For instance, the combined application of HPP and UV-C in tilapia fillets resulted in a more pronounced inhibition of microbial growth and a significant delay in detection of spoilage indicators compared to individual treatments. The combined treatment effectively reduced total aerobic counts and slowed the accumulation of TVBN, ammonia, and biogenic amines, leading to a shelf life extension of up to 2.5 times under refrigerated conditions [72]. Similarly, the integration of MAP with UV-C treatments has been shown to enhance microbial control and delay quality deterioration of tilapia fillets, extending their shelf life beyond that achieved by either treatment alone [73].

The combination of PEF, MAP achieved through direct CO_2_ flushing, and HPP in coho salmon demonstrated improved preservation of physicochemical properties, reduced enzymatic activity, and extended microbial shelf life during chilled storage. In general, mild non-thermal processes have been widely reported to preserve the fresh-like properties of fish flesh while ensuring microbial safety, as demonstrated in previous studies [74]. These findings further support the potential of integrating multiple mild preservation techniques to achieve cumulative or synergistic effects while maintaining product quality in the seafood matrices investigated [75]. However, they do not establish that similar additive or synergistic effects would occur in rehydrated cod.

The effectiveness of these combined approaches is closely linked to the interaction between the underlying mode of action of each technology. Non-thermal treatments can target different cellular components and spoilage pathways, affecting membrane integrity, genetic material, enzymatic systems, and oxidative processes. When combined, these treatments can impose multiple stresses on microbial cells, reducing their ability to recover and proliferate during storage.

However, these interactions are not always synergistic, as demonstrated in rehydrated cod [4], and may be influenced by treatment sequence, intensity, and the physicochemical characteristics of the product. In this context, the specific properties of rehydrated cod are likely to play a decisive role in determining the success of hurdle strategies.

The high-water content and the partially disrupted muscle structure may facilitate the penetration of certain antimicrobial agents, such as dissolved gases. At the same time, these characteristics may increase the susceptibility of the product to oxidative damage. Similarly, the surface characteristics of rehydrated fillets may limit the effectiveness of treatments with low penetration depth, such as UV or NTAP, unless combined with technologies capable of achieving deeper penetration into the product matrix [45,54].

These considerations indicate that future studies should test matrix-specific combinations rather than assume that strategies developed for fresh fish can be transferred directly to rehydrated cod. At present, the relative benefit of hurdle strategies over optimized individual treatments in this matrix remains unestablished.

**Figure 1:**
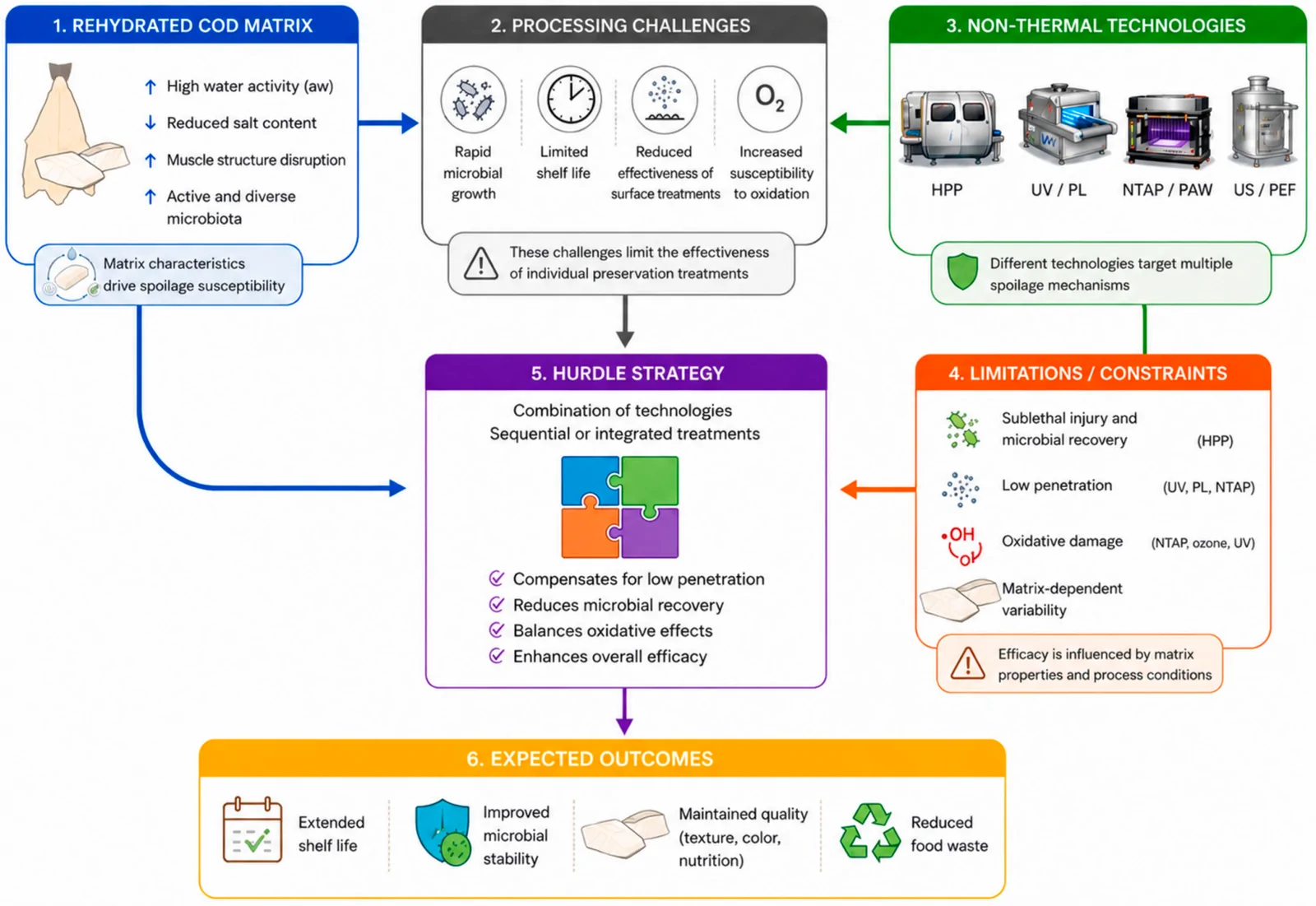
Conceptual framework illustrating the interaction between matrix characteristics, non-thermal technologies, and hurdle strategies in the preservation of rehydrated cod. **Alt text**: Rehydrated cod characteristics create preservation challenges that constrain individual non-thermal technologies, supporting tailored hurdle strategies aimed at improving microbial stability, shelf life, and product quality. Arrows indicate the conceptual relationships among the components, while colors distinguish matrix characteristics, processing challenges, non-thermal technologies, limitations, hurdle strategy, and expected outcomes.

To better illustrate the interaction between matrix characteristics, non-thermal technologies, and combined preservation approaches, a conceptual framework is proposed in Figure 1.

## 5. Industrial Perspectives and Challenges

The industrial application of non-thermal technologies to increase the shelf life of rehydrated cod presents both significant opportunities and practical challenges. The increasing demand for ready-to-cook fishery products, combined with the need to extend shelf life while preserving quality, makes rehydrated cod an attractive target for process innovation. However, the transition from laboratory-scale studies to industrial implementation remains limited.

Among the available technologies, HPP is currently the most mature in terms of industrial adoption and is already applied to different food products, including seafood, because of its ability to ensure microbial safety while largely preserving sensory attributes [76,77]. In rehydrated cod, HPP has demonstrated the potential to extend shelf life under refrigerated conditions [4,27], although its economic feasibility at industrial scale has not yet been specifically assessed. Commercial in-pack HPP systems generally have vessel capacities ranging from 35 to 525 L. For the largest 525 L units, nominal throughputs exceeding 2500 kg or L h^−1^ have been reported at 600 MPa with a 3 min holding time and approximately 50% vessel filling efficiency [78]. Capital investment has been estimated at approximately EUR 0.5–3 million, depending on vessel capacity, automation, and the number of pressure intensifiers [78]. Nevertheless, packaged solid foods are processed in batches, and actual throughput also depends on loading and unloading operations, pressurization and decompression times, holding time, package geometry, and vessel filling ratio. These constraints are particularly relevant for rehydrated cod, which requires flexible pressure-resistant packaging and uninterrupted refrigerated handling. The most established examples of industrial HPP application in the seafood sector involve molluscan shellfish. HPP is used both to reduce *Vibrio* spp. contamination and, at lower pressures, to facilitate shucking and improve meat recovery from oysters, lobsters, crabs, mussels, clams, and scallops [79]. The U.S. Food and Drug Administration (FDA) recognizes scientifically validated HPP processes as one of the approaches for controlling *Vibrio* spp. in raw molluscan shellfish while preserving raw-product characteristics [80]. These applications demonstrate the industrial maturity of HPP for specific seafood products, but do not establish its technical or economic feasibility for rehydrated cod. By comparison, industrial-scale applications of NTAP, PAW, or UV-C specifically for preserving rehydrated fish products have not been documented in the scientific literature reviewed, highlighting a significant gap in technology readiness for this product category.

Robust like-for-like cost comparisons for seafood products are not currently available. A techno-economic model for orange juice estimated minimum selling prices of USD 1.66 kg^−1^ for HPP, USD 1.64 kg^−1^ for PEF, USD 1.63 kg^−1^ for NTAP, and USD 1.62 kg^−1^ for both UV treatment and thermal pasteurization [81]. Although these estimates cannot be directly extrapolated to rehydrated cod, they illustrate the potential economic advantages of UV and NTAP over HPP and PEF, which generally require greater capital investment. However, the NTAP estimates remain subject to considerable uncertainty because commercial-scale pasteurization systems and standardized scale-up data are limited. From an operational perspective, UV and pulsed-light systems can potentially be incorporated into continuous conveyor lines, whereas NTAP throughput depends on exposure time, treatment uniformity, electrode configuration, and gas management [44,59,81]. For rehydrated cod fillets, limited penetration and surface shadowing may therefore represent more significant constraints than nominal processing speed. PAW offers an alternative approach to antimicrobial treatment. However, it still faces challenges related to limited data on food quality and the potential formation of secondary reactive compounds, the lack of standardized treatment parameters and clear regulatory pathways, and the need for cost-effective industrial scale-up [82].

Technologies such as PEF and US, although less effective as stand-alone decontamination methods, may still offer advantages in industrial contexts. In particular, their ability to enhance mass transfer or modify tissue structure could be exploited to improve process efficiency or product functionality when used in combination with other preservation strategies.

Another critical aspect for industrial implementation is packaging, since the integration of non-thermal technologies with systems such as vacuum [27] or MAP plays a key role in maintaining product stability during storage and distribution. As observed for rehydrated cod, the effectiveness of these combined approaches is not always straightforward and may depend on product-specific factors, including gas permeability, water activity, and the composition of the microbial community [4,27].

Regulatory requirements differ substantially across regions and technologies. In the European Union, HPP and other non-thermal processing technologies are generally subject to the applicable food safety and hygiene requirements. Regulation (EU) 2015/2283 on novel foods may also apply if a production process, not used for food production within the Union before 15 May 1997, gives rise to significant changes in the composition or structure of a food that affect its nutritional value, metabolism, or level of undesirable substances [83]. Ionizing radiation is regulated separately under Directives 1999/2/EC and 1999/3/EC. The EU-wide positive list established under Directive 1999/3/EC covers dried aromatic herbs, spices, and vegetable seasonings, although certain Member States have maintained national authorizations for additional food categories under the applicable transitional provisions [84,85].

In the United States, seafood processors must assess food safety hazards and establish appropriate controls under the Seafood Hazard Analysis and Critical Control Point (HACCP) framework, validating control measures where required [80]. UV radiation, pulsed light, and ozone are addressed under specific provisions of Title 21 of the Code of Federal Regulations (21 CFR 179.39, 179.41, and 173.368, respectively), which establish conditions for their use in food processing. However, their applicability to a particular seafood product still depends on the relevant regulatory provisions and the intended conditions of use, while their effectiveness as food safety controls must be supported by appropriate validation [86]. Regulatory frameworks across Asian markets are not fully harmonized, and requirements must therefore be assessed on a country-by-country basis. This is particularly evident for food irradiation, for which national authorizations differ in terms of eligible food categories and permitted doses [87]. More broadly, the absence of clear, technology-specific regulatory guidance for NTAP and PAW in many jurisdictions may hinder their industrial implementation.

Overall, non-thermal technologies differ in their level of industrial maturity and potential applicability to rehydrated cod. HPP is currently the most technologically mature option, whereas UV-C, NTAP, PAW, and other emerging approaches face greater challenges regarding treatment uniformity, scale-up, process integration, and regulatory acceptance. Their environmental benefits should not be assumed, as energy consumption and overall environmental impacts depend on equipment configuration, production scale, treatment intensity, and packaging requirements [81]. Although non-thermal technologies may contribute to preserving nutritional and sensory quality and extending product shelf life [88], potentially contributing to food waste reduction, their economic and environmental feasibility must be evaluated under conditions specific to rehydrated cod. Therefore, matrix-specific validation and optimization of treatment parameters, potentially in combination with other preservation hurdles, are needed before their suitability for industrial implementation can be established.

## 6. Future Research Directions

Despite the growing interest in non-thermal technologies for seafood preservation, several knowledge gaps remain regarding their application to rehydrated cod, and a critical lack of matrix-specific studies severely limits process optimization and the development of tailored preservation strategies. Future research should be organized around three priorities: first, standardized matrix-specific validation of microbial efficacy and quality effects; second, optimization of hurdle combinations only after effective individual treatments have been identified; and third, industrial validation addressing scalability, cost, environmental performance, regulation, and consumer acceptance.

The first priority is the systematic evaluation of non-thermal technologies directly on rehydrated cod, as most existing studies have been conducted on fresh or frozen seafood. This includes studies focused not only on microbial inactivation efficiency, but also on the impact on quality attributes such as texture, color, lipid oxidation, and sensory properties during storage.

Future research should also determine whether DPCD, electrolyzed water or ice, OMF-assisted supercooling, and bioactive coatings can provide technologically relevant benefits in rehydrated cod. Initial studies should assess CO_2_ penetration and quality changes during DPCD treatment; the influence of residual salt and organic matter on electrolyzed-water stability and antimicrobial activity; the specific contribution of OMFs independently of PEFs; and coating adhesion, release kinetics, and sensory effects on the moist product surface. Only the most promising approaches should subsequently be evaluated as complementary hurdles under industrially relevant processing and refrigerated storage conditions.

A better understanding of these matrix-dependent effects would also support the development of more effective hurdle-based preservation approaches. Future studies should investigate the combined application of different non-thermal technologies, as well as their integration with conventional preservation factors such as temperature control, salting, and packaging. Particular attention should be paid to treatment sequence, intensity, and the potential for synergistic or antagonistic interactions among preservation factors. At the same time, a deeper understanding of microbial dynamics in rehydrated cod is needed. The characterization of spoilage microbiota and their response to non-thermal treatments could provide valuable insights for the design of targeted preservation strategies. In this context, the application of advanced microbiome-based analytical approaches, such as metagenomics, could be valuable for bridging current knowledge gaps and to elucidate the mechanisms underlying microbial adaptation and post-treatment recovery.

In light of the industrial and techno-economic considerations discussed in the previous sections, it is also evident that future research should place greater emphasis on industrial scalability. Process optimization should therefore consider not only efficacy, but also sustainability, including energy efficiency, environmental impact, and economic feasibility, particularly in comparison with conventional preservation methods. The development of continuous or semi-continuous processing systems is expected to further enhance their industrial applicability.

Finally, consumer acceptance and regulatory aspects should not be overlooked. Understanding consumer perceptions of non-thermal technologies, particularly in relation to product quality and safety, is essential to support market uptake. At the same time, greater harmonization of regulatory frameworks across regions may facilitate the wider implementation of these innovative preservation approaches within the seafood sector.

## 7. Conclusions

Rehydration transforms salted cod into a highly perishable matrix whose residual salt content, altered muscle structure, and microbial ecology affect preservation performance. Among the technologies reviewed, HPP currently provides the strongest direct evidence of microbial control and shelf life extension in rehydrated cod, although treatment outcomes vary with processing and storage conditions. Evidence for the other non-thermal technologies remains largely extrapolated from different seafood matrices, while the benefits of hurdle strategies have not yet been demonstrated consistently in rehydrated cod. Consequently, neither individual nor combined treatments can currently be recommended without matrix-specific validation. Standardized studies conducted under industrially relevant conditions are needed to establish effective treatment parameters, evaluate microbial safety and product quality throughout refrigerated storage, and determine economic and regulatory feasibility.

## Figures and Tables

**Table 1:** Summary of emerging non-thermal technologies potentially applicable to rehydrated cod preservation, including their mechanisms of action, advantages, and main limitations.

*Technology*	*Evidence for Rehydrated Cod*	*Antimicrobial Mechanism of Action*	*Advantages*	***Limitations***
*HPP*	Direct evidence for microbial control and shelf-life extension	Membrane disruption, protein denaturation and enzymatic inactivation under high pressure	Effective microbial inactivation; preservation of sensory and nutritional quality; shelf life extension	High capital cost; batch processing; potential lipid oxidation; possible recovery and subsequent growth of surviving microorganisms during storage
*NTAP*	No direct evidence; extrapolated from other seafood	Oxidative damage of membranes, proteins and DNA, induced by reactive oxygen and nitrogen species	Strong antimicrobial potential; operation at near-ambient temperature; suitability for heat-sensitive foods	Limited penetration depth; risk of oxidation; lack of process standardization
*UV-C*	No direct evidence; extrapolated from other seafood	DNA damage via pyrimidine dimer formation	Low cost; residue-free; easy implementation; effective surface decontamination	Very low penetration depth; uneven exposure; potential oxidation
*PEF*	Direct evidence for rehydration and mass-transfer enhancement; no direct preservation evidence	Electroporation of cell membranes with increased permeability	Enhancement of mass transfer; minimal thermal impact	Limited antimicrobial efficacy in solid foods; mainly applicable as pre-treatment
*Ozone*	No direct evidence; extrapolated from other seafood	Oxidative damage to cellular components through strong oxidizing activity	Effective antimicrobial action; no chemical residues	Lipid oxidation; instability; need for precise dose control
*PL*	No direct evidence; extrapolated from other seafood	Photochemical, photophysical and photothermal effects induced by high-intensity light pulses	Rapid treatment; effective surface decontamination	Limited penetration depth; risk of overheating and oxidation
*US*	No direct preservation evidence; extrapolated from cod and other seafood	Acoustic cavitation causing mechanical disruption and cell membrane damage	Enhancement of mass transfer; low energy input	Limited antimicrobial efficacy; potential structural alterations
*Ionizing irradiation*	No direct evidence; extrapolated from other seafood	DNA damage through ionization and free radical formation	High microbial inactivation efficiency; shelf life extension	Regulatory constraints; consumer acceptance; potential quality changes

**Representative references:** HPP [4,9]; NTAP [29,32]; UV-C [33]; PEF [10]; ozone [34]; PL [35]; US [36,37]; ionizing irradiation [38].

## Data Availability

No new data were created or analyzed in this study. Data sharing is not applicable to this article.

## References

[1] Perdikaris S. (1999). From chiefly provisioning to commercial fishery: Long-term economic change in Arctic Norway. World Archaeol..

[2] Star B., Boessenkool S., Gondek A.T., Nikulina E.A., Hufthammer A.K., Pampoulie C., Knutsen H., André C., Nistelberger H.M., Dierking J. (2017). Ancient DNA reveals the Arctic origin of Viking Age cod from Haithabu, Germany. Proc. Natl. Acad. Sci. USA.

[3] Barat J.M., Rodríguez-Barona S., Andrés A., Visquert M. (2004). Mass transfer analysis during the cod desalting process. Food Res. Int..

[4] Rode T.M., Rotabakk B.T. (2021). Extending shelf life of desalted cod by high pressure processing. Inn. Food Sci. Emerg. Dis..

[5] Rodrigues M.J., Ho P., López-Caballero M.E., Vaz-Pires P., Nunes M.L. (2003). Characterization and identification of microflora from soaked cod and respective salted raw materials. Food Microbiol..

[6] Ekonomou S.I., Boziaris I.S. (2021). Non-thermal methods for ensuring the microbiological quality and safety of seafood. Appl. Sci..

[7] Zhao Y.M., de Alba M., Sun D.W., Tiwari B. (2019). Principles and recent applications of novel non-thermal processing technologies for the fish industry—A review. Crit. Rev. Food Sci. Nutr..

[8] Rathod N.B., Kulawik P., Ozogul Y., Ozogul F., Bekhit A.E.D.A. (2022). Recent developments in non-thermal processing for seafood and seafood products: Cold plasma, pulsed electric field and high hydrostatic pressure. Int. J. Food Sci. Technol..

[9] Fidalgo L.G., Mota M.J., D’Amil J., Queirós R.P., Pinto C.A., Delgadillo I., Saraiva J.A. (2024). Quality Changes on Cod Fish (*Gadus morhua*) during Desalting Process and Subsequent High-Pressure Pasteurization. Appl. Sci..

[10] Genovese J., Tappi S., Tylewicz U., D’Elia F., De Aguiar Saldanha Pinheiro A.C., Rocculi P. (2022). Dry-salted cod (*Gadus morhua*) rehydration assisted by pulsed electric fields: Modelling of mass transfer kinetics. J. Sci. Food Agric..

[11] Nguyen M.V., Arason S., Thorarinsdottir K.A., Thorkelsson G., Gudmundsdóttir A. (2010). Influence of salt concentration on the salting kinetics of cod loin (*Gadus morhua*) during brine salting. J. Food Eng..

[12] Thorarinsdottir K.A., Arason S., Bogason S.G., Kristbergsson K. (2004). The effects of various salt concentrations during brine curing of cod (*Gadus morhua*). Int. J. Food Sci. Technol..

[13] Brodowska A.J., Nowak A., Śmigielski K. (2018). Ozone in the food industry: Principles of ozone treatment, mechanisms of action, and applications: An overview. Crit. Rev. Food Sci. Nutr..

[14] Di Luccia A., Alviti G., Lamacchia C., Faccia M., Gambacorta G., Liuzzi V., Musso S.S. (2005). Effects of the hydration process on water-soluble proteins of preserved cod products. Food Chem..

[15] Nguyen M.V., Thorarinsdottir K.A., Gudmundsdottir A., Thorkelsson G., Arason S. (2011). The effects of salt concentration on conformational changes in cod (*Gadus morhua*) proteins during brine salting. Food Chem..

[16] Ozuna C., Puig A., García-Pérez J.V., Cárcel J.A. (2014). Ultrasonically enhanced desalting of cod (*Gadus morhua*): Mass transport kinetics and structural changes. LWT Food Sci. Technol..

[17] Lorentzen G., Ageeva T.N., Heia K. (2021). Desalting of dried salt-cured cod (*Gadus morhua* L.) without water renewal—3D imaging of volume change. Food Control.

[18] Thorarinsdottir K.A., Arason S., Sigurgisladottir S., Valsdottir T., Tornberg E. (2011). Effects of different pre-salting methods on protein aggregation during heavy salting of cod fillets. Food Chem..

[19] Martínez M., Dutil J.-D., Guderley H. (2000). Longitudinal and allometric variation in indicators of muscle metabolic capacities in Atlantic cod (*Gadus morhua*). J. Exp. Zool..

[20] Oliveira H., Gonçalves A., Nunes M.L., Vaz-Pires P., Costa R. (2016). Influence of temperature and fish thickness on the mass transfer kinetics during the cod (*Gadus morhua*) desalting process. J. Sci. Food Agric..

[21] Stoknes I.S., Walde P.M., Synnes M. (2005). Proteolytic activity in cod (*Gadus morhua*) muscle during salt curing. Food Res. Int..

[22] Bjørkevoll I., Olsen R.L., Skjerdal O.T. (2003). Origin and spoilage potential of the microbiota dominating genus Psychrobacter in sterile rehydrated salt-cured and dried salt-cured cod (*Gadus morhua*). Int. J. Food Microbiol..

[23] Fernández-Segovia I., Escriche I., Fuentes A., Serra J.A. (2007). Microbial and sensory changes during refrigerated storage of desalted cod (*Gadus morhua*) preserved by combined methods. Int. J. Food Microbiol..

[24] Oliveira H., Gonçalves A., Nunes M.L., Vaz-Pires P., Costa R. (2015). Quality changes during cod (*Gadus morhua*) desalting at different temperatures. J. Sci. Food Agric..

[25] Oliveira H., Pedro S., Nunes M.L., Costa R., Vaz-Pires P. (2012). Processing of salted cod (*Gadus* spp.): A review. Compr. Rev. Food Sci. Food Saf..

[26] Pedro S., Albuquerque M.M., Nunes M.L., Bernardo M.F. (2004). Pathogenic bacteria and indicators in salted cod (*Gadus morhua*) and desalted products at low and high temperatures. J. Aquat. Food Prod. Technol..

[27] Ferri G., Lauteri C., Scattolini M., Vergara A. (2023). Shelf Life and Safety of Vacuum Packed HPP-Treated Soaked Cod Fillets: Effects of Salt Content and Multilayer Plastic Film. Foods.

[28] Albertos I., Martín-Diana A.B., Cullen P.J., Tiwari B.K., Ojha S.K., Bourke P., Álvarez C., Rico D. (2017). Effects of dielectric barrier discharge (DBD) generated plasma on microbial reduction and quality parameters of fresh mackerel (*Scomber scombrus*) fillets. Innov. Food Sci. Emerg. Technol..

[29] Spanou A., Tzamarias A.E., Ladakis D., Koutinas A., Tsironi T. (2024). In-package cold atmospheric plasma processing for shelf-life extension of gilthead seabream (*Sparus aurata*) fillets. J. Food Sci..

[30] Pérez Alcalá D., Grande Burgos M.J., Rodríguez López J., Lucas R., Gálvez A., Pérez Pulido R. (2023). Effect of High Hydrostatic Pressure Processing on the Microbiological Quality and Bacterial Diversity of Sous-Vide-Cooked Cod. Foods.

[31] Yang Z., Xiong Q., Yin T., You J., Xiong S., Liu R. (2025). Advancements in emerging non-thermal preservation techniques for aquatic products: A comprehensive review of mechanisms, applications, and future directions. Innov. Food Sci. Emerg. Technol..

[32] Wang J., Fu T., Wang Y., Zhang J. (2022). Effects of high-voltage atmospheric cold plasma treatment on microbiological and quality characters of tilapia fillets. Foods.

[33] Tandel R.A., Solanki J.B., Tandel V.R., Patel K., Bhavika T., Tandel R.R. (2024). Ultraviolet (UV-C) radiation: An innovative non-thermal technology for quality enhancement and shelf life extension of reef cod (*Epinephelus diacanthus*) fillets during chilled storage. Int. J. Adv. Biochem. Res..

[34] Nerantzaki A., Tsiotsias A., Paleologos E.K., Savvaidis I.N., Bezirtzoglou E., Kontominas M.G. (2005). Effects of ozonation on microbiological, chemical and sensory attributes of vacuum-packaged rainbow trout stored at 4 ± 0.5 °C. Eur. Food Res. Technol..

[35] Zhang J., Zhou G., Ji S., Zou L., Liang J. (2022). Effect of pulse light on the quality of refrigerated (4 °C) large yellow croaker (*Pseudosciaena crocea*). LWT.

[36] Pedrós-Garrido S., Condón-Abanto S., Beltrán J.A., Lyng J.G., Brunton N.P., Bolton D., Whyte P. (2017). Assessment of high intensity ultrasound for surface decontamination of salmon (*S. salar*), mackerel (*S. scombrus*), cod (*G. morhua*) and hake (*M. merluccius*) fillets, and its impact on fish quality. Innov. Food Sci. Emerg. Technol..

[37] Dong X., Raghavan V. (2024). High-intensity ultrasound treatment of Atlantic cod: Impact on nutrients, structure, sensory quality, bioactivity, and in-vitro digestibility. Food Res. Int..

[38] Yu H., Zhang J., Li H., Zhao Y., Xia S., Qiu Y., Zhu J. (2022). Effects of E-beam irradiation on the physicochemical properties of Atlantic cod (*Gadus morhua*). Food Biosci..

[39] Koutsoumanis K., Alvarez-Ordóñez A., Bolton D., Bover-Cid S., Chemaly M., Davies R., De Cesare A., Herman L., Hilbert F., EFSA Panel on Biological Hazards (BIOHAZ) (2022). The efficacy and safety of high-pressure processing of food. EFSA J..

[40] Ekonomou S.I., Bulut S., Karatzas K.A.G., Boziaris I.S. (2020). Inactivation of Listeria monocytogenes in raw and hot smoked trout fillets by high hydrostatic pressure processing combined with liquid smoke and freezing. Innov. Food Sci. Emerg. Technol..

[41] Zhang Y., Bi Y., Wang Q., Cheng K.W., Chen F. (2019). Application of high pressure processing to improve digestibility, reduce allergenicity, and avoid protein oxidation in cod (*Gadus morhua*). Food Chem..

[42] De Aguiar Saldanha Pinheiro A.C., Tappi S., Soglia F., Baldi G., Molina Hernandez J.B., Laghi L., Dalla Rosa M., Petracci M., Rocculi P. (2025). Effect of high hydrostatic pressure (HHP) on proteins, lipids, and water state of three different thawed seafood products. Ital. J. Food Sci..

[43] Bolumar T., Orlien V., Sikes A., Aganovic K., Bak K.H., Guyon C., Stübler A.S., de Lamballerie M., Hertel C., Brüggemann D.A. (2021). High-pressure processing of meat: Molecular impacts and industrial applications. Compr. Rev. Food Sci. Food Saf..

[44] López M., Calvo T., Prieto M., Múgica-Vidal R., Muro-Fraguas I., Alba-Elías F., Alvarez-Ordóñez A. (2019). A review on non-thermal atmospheric plasma for food preservation: Mode of action, determinants of effectiveness, and applications. Front. Microbiol..

[45] Shishir M.R.I., Kamal M.M., Karim N., Saifullah M., Khan S., Zhang K., Marappan G., Aalim H., Hashim S.B.H., Zhai X. (2025). Cold plasma and integrated approaches for fresh produce preservation: Mechanisms, quality attributes, challenges, and future directions. Trends Food Sci. Technol..

[46] Sasikumar R., Selva Kumar T., Mangang I.B., Kaviarasu G., Kaushik R., Mansingh P., Tomer V., Jaiswal A.K. (2025). A comprehensive review on cold plasma applications in the food industry. Sustain. Food Technol..

[47] Colejo S., Alvarez-Ordóñez A., Prieto M., González-Raurich M., López M. (2018). Evaluation of ultraviolet light (UV), non-thermal atmospheric plasma (NTAP) and their combination for the control of foodborne pathogens in smoked salmon and their effect on quality attributes. Innov. Food Sci. Emerg. Technol..

[48] Pérez-Andrés J.M., de Alba M., Harrison S.M., Brunton N.P., Cullen P.J., Tiwari B.K. (2020). Effects of cold atmospheric plasma on mackerel lipid and protein oxidation during storage. LWT.

[49] Liu C., Wan J., Wang Y., Chen G. (2023). Effects of Cold Plasma Treatment Conditions on the Lipid Oxidation Kinetics of Tilapia Fillets. Foods.

[50] Oliinychenko Y.K., Ekonomou S.I., Tiwari B.K., Stratakos A.C. (2024). Assessing the effects of cold atmospheric plasma on the natural microbiota and quality of pork during storage. Foods.

[51] Oliveira M., Fernández-Gómez P., Álvarez-Ordóñez A., Prieto M., López M. (2022). Plasma-activated water: A cutting-edge technology driving innovation in the food industry. Food Res. Int..

[52] Srivastava S., Akbar U., Mondol M.S.A., Dash K.K., Junaid P.M., Manzoor M., Khan S.A., Dar A.H. (2025). Plasma Activated Water in Enhancing Food Safety and Quality: A Comprehensive Review. Plasma Chem. Plasma Process..

[53] Jadhav H.B., Annapure U.S., Deshmukh R.R. (2021). Non-thermal Technologies for Food Processing. Front. Nutr..

[54] Maioto R., Santos S., Dias A.A., Aires C., Inês A., Sedrine N.B., Mendes P., Rodrigues P., Sampaio A. (2026). UV Radiation: Applications on Surfaces in the Food Industry. Appl. Sci..

[55] Pankyamma V., Rao B.M., Preethi G.K., Debbarma J. (2025). Effects of UV-C Radiation on Controlling the Biochemical and Microbial Spoilage of Dried Fish During Storage. J. Aquat. Food Prod. Technol..

[56] Andoni E., Ozuni E., Bijo B., Shehu F., Branciari R., Miraglia D., Ranucci D. (2021). Efficacy of Non-thermal Processing Methods to Prevent Fish Spoilage. J. Aquat. Food Prod. Technol..

[57] Chawla A., Bell J.W., Janes M.E. (2007). Optimization of Ozonated Water Treatment of Wild- Caught and Mechanically Peeled Shrimp Meat. J. Aquat. Food Prod. Technol..

[58] Gelman A., Sachs O., Khanin Y., Drabkin V. (2005). Effect of Ozone Pretreatment on Fish Storage Life at Low Temperatures. J. Food Prot..

[59] Mandal R., Mohammadi X., Wiktor A., Singh A., Singh A.P. (2020). Applications of Pulsed Light Decontamination Technology in Food Processing: An Overview. Appl. Sci..

[60] Han G., Wang Y., Li K., Liu T., Gong L., Liu W., Xu X. (2025). Effect of pulsed light treatment on 4 ◦ C refrigerated Atlantic salmon (*Salmo salar*) quality: Shelf life, microbial community changes, and antimicrobial mechanisms. Food Control.

[61] Annamalai J., Murthy N.L., Behere G.S. (2025). Irradiation Techniques for Shelf Life Extension of Seafood: An Overview. Eur. J. Nutr. Food Saf..

[62] Kuzmin S.V., Esaulova O.V., Moshchenskaya N.V., Skopin A.Y., Balakaeva A.V., Rusakov V.N., Barvina A.Y. (2025). The effectiveness of using innovative technologies of ionizing radiation for disinfection of fish products. Fisheries.

[63] Perez Cenci M., Marchetti M.D., Felix M.L., Cova M.C., Garcia Loredo A.B., Tomac A. (2025). Screening the effect of ionizing radiation on microbiological quality, sensory acceptability and shelf life extension of lean fish fillets. Radiat. Phys. Chem..

[64] Sireesha T., Gowda N.A.N., Kambhampati V. (2022). Ultrasonication in seafood processing and preservation: A comprehensive review. Appl. Food Res..

[65] Xiao W., Geng Y., Gao W., Zeng X.A., Woo M., Ma J., Han Z. (2026). A comprehensive review of the applications of pulsed electric field: Primary emphasis on extraction, modification, and inactivation. Innov. Food Sci. Emerg. Technol..

[66] Arbal A., Ghangale D., Wadje P., Kumar M.K., Byresh T.S., Sengar A.S., Sunil C.K. (2024). Dense phase carbon dioxide (DPCD) inactivation of microorganisms and enzymes, and its application in food: A review. Food Chem. Adv..

[67] Barbosa J., Puton B.M.S., Fischer B., Junges A., Paroul N., Steffens C., Zeni J., Steffens J., Valduga E., Backes G.T. (2020). Effect of supercritical CO_2_ on physicochemical characteristics and D-value of *S. aureus* in raw salmon. Ind. Biotechnol..

[68] Xuan X.T., Fan Y.F., Ling J.G., Hu Y.Q., Liu D.H., Chen S.G., Ye X.Q., Ding T. (2017). Preservation of squid by slightly acidic electrolyzed water ice. Food Control.

[69] Zhong J., Deng H., Lin N., Zheng M., Wu J., Guo Q., Pan S. (2025). Effect of slightly acidic electrolyzed water combined with nano-bubble sterilization on quality of Larimichthys crocea during refrigerated storage. Foods.

[70] Lee D., Tang J., Lee S.H., Jun S. (2024). Effect of oscillating magnetic fields (OMFs) and pulsed electric fields (PEFs) on supercooling preservation of Atlantic salmon (*Salmo salar* L.) Fillets. Foods.

[71] Khorami F., Babaei S., Valizadeh S., Naseri M., Golmakani M.-T. (2024). Bilayer coatings for extension of the shelf life of fish fillets: Incorporating seaweed sulfated polysaccharides in chitosan–alginate LbL structures. Food Sci. Nutr..

[72] Monteiro M.L.G., Mársico E.T., Mano S.B., Da Silveira Alvares T., Rosenthal A., Lemos M., Ferrari E., Lázaro C.A., Conte C.A. (2018). Combined effect of high hydrostatic pressure and ultraviolet radiation on quality parameters of refrigerated vacuum-packed tilapia (*Oreochromis niloticus*) fillets. Sci. Rep..

[73] Lázaro C.A., Monteiro M.L.G., Conte-Junior C.A. (2020). Combined effect of modified atmosphere packaging and UV-C radiation on pathogens reduction, biogenic amines, and shelf life of refrigerated tilapia (*Oreochromis niloticus*) fillets. Molecules.

[74] Jiang W., Wu-Chen R.A., Sun K., Jin S. (2025). Innovations in non-thermal processing technologies for preserving the nutritional value and quality of fish meat: A review. Int. J. Food Sci. Technol..

[75] Pérez-Won M., Cepero-Betancourt Y., Reyes-Parra J.E., Palma-Acevedo A., Tabilo-Munizaga G., Roco T., Aubourg S.P., Lemus-Mondaca R. (2021). Combined PEF, CO_2_ and HP application to chilled coho salmon and its effects on quality attributes under different rigor conditions. Innov. Food Sci. Emerg. Technol..

[76] Serment-Moreno V., Queirós R., Farkas D., Tonello C. (2025). Food Commercialization with High-Pressure Processing (HPP). Nonthermal Processing Technologies for Food.

[77] Kotadiya D.P., Borichangar R.V., Tandel S.K., Parmar J.N., Varma R.B., Sapra D.H. (2025). A Review on High-pressure Processing for Seafood Preservation. J. Biochem. Int..

[78] Houška M., Silva F.V.M., Evelyn B.R., Terefe N.S., Tonello C. (2022). High pressure processing applications in plant foods. Foods.

[79] Roobab U., Fidalgo L.G., Arshad R.N., Khan A.W., Zeng X.A., Bhat Z.F., Bekhit A.E.D.A., Batool Z., Aadil R.M. (2022). High-pressure processing of fish and shellfish products: Safety, quality, and research prospects. Compr. Rev. Food Sci. Food Saf..

[80] U.S. Food and Drug Administration (2022). Fish and Fishery Products Hazards and Controls Guidance.

[81] Yang M., Wang Q. (2025). Carbon footprint and cost analysis of non-thermal food processing technologies: A review with a case study on orange juice. Front. Sustain. Food Syst..

[82] Arumugam D., Li S., Rao M., Peng R., Liu H., Huo B., Wei S., Wang J. (2026). Non-thermal technologies for seafood preservation and shelf-life extension: A detailed review. Food Qual. Saf..

[83] European Parliament and Council of the European Union (2015). Regulation (EU) 2015/2283 of 25 November 2015 on novel foods. Off. J. Eur. Union.

[84] European Parliament and Council of the European Union (1999). Directive 1999/2/EC on foods and food ingredients treated with ionising radiation. Off. J. Eur. Communities.

[85] European Parliament and Council of the European Union (1999). Directive 1999/3/EC establishing a Community list of foods and food ingredients treated with ionising radiation. Off. J. Eur. Communities.

[86] Electronic Code of Federal Regulations (2026). Title 21: Food and Drugs, Sections 173.368, 179.39, and 179.41. https://www.ecfr.gov/current/title-21.

[87] International Atomic Energy Agency (2021). Irradiation and Trade in Food and Agricultural Products.

[88] Younis K., Jahan K., Qadri O.S., Osama K., Yousuf O. (2025). Non-thermal processing techniques for sustainable food supply chains. Sustain. Futures.

